# Preterm birth and small for gestational age in relation to alcohol consumption during pregnancy: stronger associations among vulnerable women? results from two large Western-European studies

**DOI:** 10.1186/1471-2393-13-49

**Published:** 2013-02-22

**Authors:** Manuela Pfinder, Anton E Kunst, Reinhold Feldmann, Manon van Eijsden, Tanja G M Vrijkotte

**Affiliations:** 1 Bielefeld Graduate School in History and Sociology, Faculty of Sociology, Bielefeld University, PO Box 100131, 33501 Bielefeld, Germany; 2 Department of Public Health, Academic Medical Centre, University of Amsterdam, Meibergdreef 9, 1105 AZ, Amsterdam, The Netherlands; 3 FAS Ambulance, Polyclinic for Children’s and Youth Medicine, University Hospital Munster, Albert-Schweitzer-Straße 33, 48129 Munster, Germany; 4 Department of Epidemiology, Documentation and Health Promotion, Public Health Service of Amsterdam (GGD), Nieuwe Achtergracht 100, 1018 WT Amsterdam, PO Box 22001000 CE, Amsterdam, The Netherlands; 5 Department of Health Sciences, VU University, De Boelelaan 1085, 1081 HV, Amsterdam,The Netherlands

**Keywords:** Prenatal alcohol exposure, Pregnancy outcomes, Maternal education, Distress, Smoking

## Abstract

**Background:**

Inconsistent data on the association between prenatal alcohol exposure and a range of pregnancy outcomes, such as preterm birth (PTB) and small for gestational age (SGA) raise new questions. This study aimed to assess whether the association between low-moderate prenatal alcohol exposure and PTB and SGA differs according to maternal education, maternal mental distress or maternal smoking.

**Methods:**

The Amsterdam Born Children and their Development (ABCD) Study (N = 5,238) and the German Health Interview and Examination Survey for Children and Adolescents (KiGGS) (N = 16,301) are both large studies. Women provide information on alcohol intake in early pregnancy, 3 months postpartum and up to 17 years retrospectively. Multivariate logistic regression analyses and stratified regression analyses were performed to examine the association between prenatal alcohol exposure and PTB and SGA, respectively.

**Results:**

No association was found between any level of prenatal alcohol exposure (non-daily, daily, non-abstaining) and SGA. The offspring of daily drinkers and non-abstainers had a lower risk of PTB [ABCD: odds ratio (OR) 0.31, 95% confidence interval (CI) 0.13, 0.77; KiGGS: OR 0.75, 95% CI 0.57, 0.99]. Interactions with maternal education, maternal distress or maternal smoking were not significant.

**Conclusions:**

Although these results should be interpreted with caution, both studies showed no adverse effects of low-moderate prenatal alcohol exposure on PTB and SGA, not even in the offspring of women who were disadvantaged in terms of low education, high levels of distress, or smoking during pregnancy.

## Background

For over 40 years, there have been reports on associations between high levels of maternal alcohol intake during pregnancy and various adverse birth outcomes including fetal alcohol syndrome (FAS) [1,2]. A link has been shown between maternal alcohol intake during pregnancy and small for gestational age (SGA) and preterm birth (PTB) [3-23]. A recent meta-analysis found that the risk of SGA and PTB was not increased in mothers who drank alcohol during pregnancy on low to moderate levels [20]. However, studies included in that meta-analysis and a recent systematic review [7] suggest that the reported effects of low to moderate alcohol consumption on PTB and SGA are inconsistent across studies.

One reason for this inconsistency could be differences in the studied populations with respect to socioeconomic characteristics. There is evidence that adverse effects of high prenatal alcohol exposure are more likely to occur in the offspring of women with a lower socioeconomic status (SES) than of those with a higher SES [1,2,7,20,24-29]. This suggests that alcohol intake during pregnancy is more likely to have an impact on PTB and SGA in the offspring of women with a low SES.

A reason for this stronger effect of alcohol exposure in the offspring of low SES women could be the interaction with smoking and maternal distress. These factors are reported to be more prevalent in women with a lower SES, also during pregnancy [30]. Smoking and stress could create a biological environment in which alcohol shows more disadvantageous effects due to interactive mechanisms [5,22,26,27,31-37].

Animal studies have shown that stress causes an increased release of hormones that interact with alcohol (such as corticosterone) and that this interaction between stress and alcohol affects birth weight and brain development [36]. Studies on rhesus monkeys showed that prenatal exposure to stress enlarges the effect of prenatal alcohol exposure on brain development, especially in the dopaminergic system [37]. In humans, however, evidence for effect modification by maternal distress is lacking.

Moreover, smoking during pregnancy was found to exacerbate the impact of alcohol on health outcomes as exposure to tobacco smoke causes reduced blood oxygen content, reduced blood flow and decreased levels of growth stimulating nutrients [26,27]. In humans, studies from the USA [5], England [31], the Netherlands [22] and Denmark [32] suggest more adverse effects of alcohol on SGA and low birth weight in the offspring of mothers who drank and smoked during pregnancy compared to those who only drank.

The present study investigates the associations between low to moderate alcohol consumption and two important perinatal outcomes (i.e. PTB and SGA) and assesses whether these associations are modified by levels of maternal education, maternal mental distress or maternal smoking during pregnancy.

## Methods

This study used data from two studies with large samples that enabled stratification by mothers’ characteristics. These two studies are the Amsterdam Born Children and their Development (ABCD) study and the German Health Interview and Examination Survey for Children and Adolescents (KiGGS).

### ABCD study (Netherlands)

The ABCD study is described in detail elsewhere [38]. Briefly, this large multiethnic population-based cohort study was established in 2003. A total of 12,373 pregnant women were approached and finally 8,266 pregnant women (response rate 67%) who live in Amsterdam were interviewed between January 2003 and March 2004 within two weeks after their first pregnancy examination (on average in the 16th week of gestation). A registration form, including a range of personal data, was filled in by the care provider. Of the 8,266 women surveyed, 7,863 gave birth to viable singletons and 132 gave birth to viable multiples. Between four and seven days after delivery, nurses examined the health status of the infants. Nurses also reported on perinatal outcomes, including the date of birth, birth weight, gender, gestational age, and screenings on congenital metabolic disorders. Further data on the perinatal outcome were received from the Dutch Perinatal Registration [39].

Within the third month after giving birth, the 6,854 mothers who gave permission for a follow-up received an infant questionnaire on the course of the pregnancy, the delivery, the health of the baby, its growth and development, and the maternal lifestyle during and after pregnancy. A total of 5,218 mothers filled in the infant questionnaire (response rate 76%).

### KiGGS study (Germany)

The KiGGS study is the first nationwide survey in Germany on the health of children and adolescents aged 0–17 years, implemented by the Robert Koch Institute (RKI) and commissioned by the German Federal Ministry of Health. Details on the survey can be found elsewhere [40]. Briefly, the cross-sectional study was implemented in 167 sample points throughout Germany with an oversampling of children and adolescents with a migrant background and an oversampling of those from Eastern Germany. Eligible children and adolescents were born between 1985 and 2006 with their main residence in Germany. Participants were enrolled between May 2003 and May 2006 (median 31 December 2004). The survey includes age-appropriate questionnaires to be filled out by the parents and questionnaires to be filled out by the children and adolescents aged 11–17 years. The questionnaires cover topics of general mental and somatic health, sense of well-being, social environment, living conditions, family structures and socio-demographics. The children and adolescents took part in physical examinations and tests. At central laboratories blood and urine samples were taken, and a computer-assisted personal interview (CAPI) was performed by a physician. Of the total sample of 28,299 participants, 17,641 children and adolescents in the age range 0–17 and their parents could be surveyed (response rate 66.6%).

### Alcohol consumption during pregnancy and study population

In the KiGGS study, alcohol consumption during pregnancy was measured retrospectively by self-reports, with the question “Did the mother consume alcohol during pregnancy?” Possible answer categories were “no”, “moderately”, or “regularly”.

In the ABCD study, maternal alcohol intake was measured (on average) at the 16th week of gestation within the pregnancy questionnaire by asking about the alcohol intake in the previous week. Answer possibilities were “no; yes, but less than one glass per day on average; yes, on average … glasses per day”. Three months after delivery (mean 13.6 weeks) the women were again asked about their alcohol intake during pregnancy within the infancy questionnaire by the question “Did you drink alcohol during the pregnancy?” Answer categories were “no” or “yes”. In case the women gave a positive answer on alcohol intake during pregnancy, they were asked “How much did you drink during the last month of the pregnancy?” For an answer, they were asked to specify the number of glasses drunk (on average) per day.

Information on alcohol intake in early pregnancy was restricted to the week prior to administration of the questionnaire. However, we were able to identify part of the women who consumed alcohol in early pregnancy, although not in the week prior to the interview, by means of retrospective questions on alcohol intake from the postpartum survey.

In our sample of the KiGGS study, the number of self-reported regular drinkers during pregnancy was very low (N = 9). In addition, the information on ‘moderate’ and ‘regular’ alcohol intake during pregnancy is based on self-reports and, thus, it is influenced by the respondents’ subjective evaluation of the quantities ‘moderate’ and ‘regular’. In order to avoid the difficulty of distinguishing between ‘moderate’ and ‘regular’ alcohol intake, we have classified the mothers into the broad groups of ‘abstainers’ and ‘non-abstainers’.

In the ABCD study, we combined the information from the pregnancy questionnaire with that from the infancy questionnaire to get a complete measurement on alcohol intake during the entire pregnancy. A variable was constructed including abstainers (reporting abstaining during pregnancy at both measurement points), non-daily drinkers (reporting at least at one measurement point that they were drinking alcohol, but not every day) and daily drinkers (reporting at least at one measurement point that they were drinking alcohol daily).

For the baseline samples we excluded twin or multiple births, births before the 24th week of gestation, non-spontaneous pregnancies, those without information on SGA, non-biological parents responding to the questionnaire and those without information on alcohol intake during pregnancy, leaving a study population of 5,238 mother-child dyads in the ABCD study and 16,301 participants in the KiGGS study.

### Outcome measures

The present study focused on SGA and PTB. SGA was defined as a birth weight below the 10th percentile for gestational age, standardised by gender and parity according to the Dutch guidelines [41]. PTB was defined as a delivery between 24 and 36 weeks plus 6 days of gestational age.

In the ABCD study, data on gestational age, (based on ultrasound or, if unavailable, onset of most recent menstrual period) and birth weight, as recorded by the obstetric care providers, were obtained from the Youth Health Department at the Municipal Health Service Amsterdam.

In the KiGGS study, gestational age and birth weight were self-reported by the parents.

### Covariates

The ABCD study and the KiGGS study measured the following covariates: maternal age, parity (0, ≥ 1), ethnicity based on maternal country of birth (ABCD: Dutch, Creole, Mediterranean, Others, KiGGS: German, Non-German), maternal (pre-pregnancy) body mass index (BMI: based on self-reported height and weight before pregnancy), hypertensive disorders (chronic hypertension and gestational hypertension), maternal education (self-reported; ABCD study: years after primary school; KiGGS study: school leaving qualifications), maternal height, maternal smoking during pregnancy (self-reported: yes, no) and maternal mental distress (in the ABCD study only).

In the ABCD study, low maternal education was defined as < 6 years of education after primary school, and mid-high maternal education as ≥ 6 years.

In the KiGGS study, low maternal education was defined as no graduation, not yet graduated or graduation from junior high school, and mid-high education was defined as graduation from the intermediate school, high school or advanced (poly) technical school.

In the ABCD study, maternal mental distress was assessed on the basis of anxiety symptoms, measured by the validated State-Trait Anxiety Inventory (STAI) [42], and depressive symptoms using the validated Dutch version of the Center for Epidemiological Studies Depression Scale (CES-D) [43]. Briefly, the STAI is a 20-item questionnaire that is meant to be administered by self reports, and that is widely used to assess anxiety during pregnancy and postpartum. The CES-D is a 20-item self-report questionnaire to detect depressive symptomatologies, but not clinical or chronic depression. However, as the CES-D scores correlate well with clinical assessments of depression [43,44] this measure is broadly used to detect high-risk groups and possible cases of depression. In our sample, the STAI and the CES-D were reliable measurements (Cronbach’s α: 0.69 and 0.94 for depression and anxiety, respectively). The total scores on depression and anxiety were highly correlated (ρ = 0.615, p < 0.001). The variables were dichotomized to correspond with the cut-off-points that showed high accuracy (0.87) in previous studies [45,46] (Likelihood Ratio of 6.8; sensitivity = 0.82; specificity = 0.88): ≥23 for high levels of depression and ≥54 for high levels of anxiety. Maternal mental distress was defined when the mother scored high on one or both scales. Maternal mental distress could not be measured in the KIGGS study.

### Statistical analyses

In descriptive statistics, one-way-analysis of variance (ANOVA) was applied to test trends in continuous factors while the Chi-squared test was applied to categorical factors. Multivariate logistic regression analyses were used to calculate the odds ratio (OR) and the 95% confidence interval (CI) that expresses the association of SGA and PTB with levels of maternal alcohol intake (abstainers were reference). In the regression analysis, the full model was adjusted for maternal age, parity, ethnicity, maternal pre-pregnancy BMI, maternal education, maternal height, smoking during pregnancy and hypertension and in the ABCD study we additionally adjusted for maternal mental distress.

In the next step, the model included interaction terms between maternal alcohol intake and, respectively, the level of maternal education, maternal smoking during pregnancy and maternal mental distress. By means of these terms, we assessed whether an interaction could be demonstrated with conventional levels of significance (p < 0.05). In addition, we fitted the full regression models for subgroups of women stratified according to maternal education, smoking during pregnancy, or maternal mental distress. This stratified analysis was added in order to describe potential interactions in terms of their direction and magnitude, and not only in terms of statistical significance. The Statistical Package of Social Sciences (SPSS) version 19.0 was used for all statistical analyses.

The KiGGS study was approved by the ethics committee of the Charité/Universitätsmedizin Berlin (Germany) and the Federal Office for the Protection of Data on 20 February 2003. Written informed consent according to the Helsinki Declaration was obtained from the participants and their parents or guardians before the subjects entered the study. For the ABCD study, the Central Committee on Research Involving Human Subjects in the Netherlands, the medical ethics review committees of the participating hospitals, and the Registration Committee of the Municipality of Amsterdam (the Netherlands) approved the protocols on 29 March 2002. All women participating in the ABCD study gave written informed consent.

## Results

### ABCD study

Of the 5,238 women in the sample, 36.2% reported non-daily alcohol intake and 5.6% reported daily alcohol intake during pregnancy (Table 1). Educational level, maternal height and maternal age were highest among daily drinkers and lowest among abstainers. The prevalence of pregnancy hypertension and pre-existing hypertension was highest among abstainers and lowest among daily drinkers (for all differences p < 0.001). The prevalence of smoking during pregnancy and mental distress was highest in the daily drinking group. Dutch women were overrepresented in the non-daily and daily drinking group whereas women with a different ethnic background were mostly abstainers (Table 1). Prevalence rates were 8.6% for SGA and 4.8% for PTB.

**Table 1:** ABCD study: sample characteristics (N = 5238) according to the level of alcohol intake during pregnancy

	**Total N = 5238**	**Abstainer N = 3049 (58.2%)**	**Non-daily drinker N = 1894 (36.2%)**	**Daily drinker N = 295 (5.6%)**	**P for trend**
Maternal education (years)	9.6 (3.7)	8.7 (3.9)	10.8 (3.0)	10.8 (3.3)	<0.001
Smoking during pregnancy (% yes)	9.4	9.1	8.8	16.9	0.013
Maternal height (cm)	169.2 (7.1)	168.1 (7.3)	170.7 (6.5)	170.7 (6.5)	<0.001
Maternal BMI (kg/m^2^)	22.8 (3.8)	23.3 (4.2)	22.2 (3.1)	22.3 (3.2)	<0.001
Maternal age (years)	31.4 (4.8)	30.5 (5.3)	32.5 (3.6)	32.9 (3.9)	<0.001
Parity (% >0)	42.8	42.4	42.7	47.1	0.280
Hypertensive disorders (% yes)					
Pregnancy hypertension	9.8	11.1	8.1	7.5	<0.001
Preexisting hypertension	3.4	3.8	3.0	2.4	
Mental distress (% high)	15.5	17.0	13.2	15.0	0.003
Ethnicity (%)					
Dutch	72.2	65.3	82.0	80.0	<0.001
Mediterranean	6.5	8.0	4.4	4.7	
Creole	6.0	10.1	0.1	1.0	
Other	15.3	16.5	13.5	14.2	

Data were missing for maternal education (N = 29), smoking during pregnancy (N = 1), mental distress (N = 28), ethnicity (N = 2).

Values are percentages for categorical factors, or means (with standard deviations) for continuous factors.

In both, the univariate and multivariate regression, neither non-daily nor daily maternal alcohol consumption was found to be related to SGA: adjusted OR for non-daily alcohol exposure was 0.88 (95% CI 0.63, 1.24); adjusted OR for daily alcohol exposure was 1.08 (95% CI 0.59, 1.97) (Table 2). On the other hand, an inverse relationship was found between alcohol consumption and the risk of PTB. After adjustment for confounders, the associations between daily alcohol intake were significant with an OR for PTB of 0.31 (95% CI 0.13, 0.77) (Table 2).

**Table 2:** Odds ratios (95% CI) and prevalence percentages of SGA and PTB and maternal alcohol intake during pregnancy

	**ABCD study**			**KiGGS study**	
	**Abstainer**	**Non-daily drinker**	**Daily drinker**	**Abstainer**	**Non-abstainer**
	**N = 3049**	**N = 1894**	**N = 295**	**N = 14089**	**N = 2012**
**SGA**					
Prevalence	9.0	8.3	6.8	9.5	9.5
Crude Model	1.00	0.94 (0.76, 1.15)	0.72 (0.44, 1.16)	1.00	1.01 (0.84, 1.21)
Full Model	1.00	1.14 (0.91, 1.43)	0.77 (0.47, 1.27)	1.00	0.98 (0.81, 1.19)
**PTB**					
Prevalence	5.5	4.1	1.7	6.5	4.8
Crude Model	1.00	0.73 (0.56, 0.97)	0.30 (0.12, 0.73)	1.00	0.71 (0.54, 0.93)
Full Model	1.00	0.77 (0.58, 1.04)	0.31 (0.13, 0.77)	1.00	0.75 (0.57, 0.99)

Full Model: adjusted for education, smoking during pregnancy, maternal height, parity, BMI, maternal age, ethnicity, maternal distress (in ABCD study only) and hypertension (in ABCD study only).

For all analyses on SGA, parity was excluded, because SGA is corrected for parity by definition.

*CI* = confidence interval; *SGA* = small for gestational age; *PTB* = preterm birth;

The interaction term between alcohol intake during pregnancy and maternal education was not significant (p-values for interaction ranged from 0.104 to 0.669). The same applied to smoking during pregnancy and mental distress (p-values for interaction ranged from 0.204 to 0.871).

Stratified analysis by educational level yielded no evidence to suggest that alcohol consumption would have an adverse effect on SGA or PTB within the lower educated group (Table 3). Stratified analyses by smoking showed that the positive association between non-daily and daily alcohol intake during pregnancy and PTB was only detectable in non-smokers. In smokers, the positive effect of alcohol on PTB disappeared (Table 4). Stratification by maternal mental distress suggested that the inverse association between alcohol consumption and PTB was stronger among women with low levels of stress (Table 5).

**Table 3:** Odds ratios (95% CI) from the full model of SGA and PTB and maternal alcohol intake during pregnancy, categorized by maternal education level

	**ABCD study**				**KiGGS study**		
	**(%)**	**Abstainer**	**Non-daily drinker**	**Daily drinker**	**(%)**	**Abstainer**	**Non-abstainer**
		**N = 3049**	**N = 1894**	**N = 295**		**N = 14089**	**N = 2012**
**SGA**							
Mid-high education	7.7	1.00	1.18 (0.92, 1.51)	0.84 (0.50, 1.41)	9.0	1.00	1.08 (1.06, 1.09)
Low education	13.7	1.00	0.96 (0.53, 1.74)	0.28 (0.04, 2.13)	11.4	1.00	0.72 (0.48, 1.08)
**PTB**							
Mid-high education	4.5	1.00	0.79 (0.58, 1.07)	0.37 (0.15, 0.92)	5.3	1.00	0.77 (0.57, 1.03)
Low education	5.9	1.00	0.84 (0.35, 1.01)	---	6.4	1.00	0.57 (0.26, 1.24)

Full Model: adjusted for smoking during pregnancy, maternal height, parity, *BMI*, maternal age, ethnicity, maternal distress (in ABCD study only) and hypertension (in ABCD study only).

For all analyses on SGA, parity was excluded, because SGA is corrected for parity by definition.

*CI* = confidence interval; *SGA* = small for gestational age; *PTB* = preterm birth;

**Table 4:** Odds ratios (95% CI) from the full model of SGA and PTB and maternal alcohol intake during pregnancy, categorized by smoking during pregnancy

	**ABCD study**				**KiGGS study**		
	**(%)**	**Abstainer**	**Non-daily drinker**	**Daily drinker**	**(%)**	**Abstainer**	**Non-abstainer**
		**N = 3049**	**N = 1894**	**N = 295**		**N = 14089**	**N = 2012**
**SGA**							
Non-smoker	7.8	1.00	1.17 (0.91, 1.50)	0.76 (0.41, 1.39)	8.1	1.00	1.06 (0.85, 1.33)
Smoker	16.6	1.00	1.00 (0.56, 1.77)	0.77 (0.31, 1.91)	16.6	1.00	0.80 (0.56, 1.14)
**PTB**							
Non-smoker	4.6	1.00	0.75 (0.55, 1.03)	0.16 (0.04, 0.64)	6.1	1.00	0.80 (0.60, 1.08)
Smoker	6.5	1.00	1.00 (0.42, 2.42)	0.85 (0.22, 3.34)	6.9	1.00	0.56 (0.26, 1.19)

Full Model: adjusted for education, maternal height, parity, *BMI*, maternal age, ethnicity, maternal distress (in ABCD study only) and hypertension (in ABCD study only).

For all analyses on SGA, parity was excluded, because SGA is corrected for parity by definition.

*CI* = confidence interval; *SGA* = small for gestational age; *PTB* = preterm birth;

**Table 5:** Odds ratios (95% CI) from the full model of SGA and PTB and maternal alcohol intake during pregnancy, categorized by maternal mental distress

	**ABCD study**			
	**(%)**	**Abstainer**	**Non-daily drinker**	**Daily drinker**
		**N = 3049**	**N = 1894**	**N = 295**
**SGA**				
No distress	8.2	1.00	1.12 (0.88, 1.42)	0.67 (0.38, 1.19)
Distress	11.3	1.00	1.40 (0.72, 2.69)	1.68 (0.55, 5.08)
**PTB**				
No distress	4.5	1.00	0.75 (0.55, 1.03)	0.23 (0.07, 0.72)
Distress	7.0	1.00	1.01 (0.46, 2.24)	0.68 (0.14, 3.23)

Full Model: adjusted for education, smoking during pregnancy, maternal height, parity, BMI, maternal age, ethnicity and hypertension.

For all analyses on SGA, parity was excluded, because SGA is corrected for parity by definition.

*CI* = confidence interval; *SGA* = small for gestational age; *PTB* = preterm birth;

### KiGGS study

Of the 16,301 women in the baseline sample, 13.6% reported drinking alcohol during pregnancy. Educational level, height, parity, age at birth of the child and the prevalence of smoking during pregnancy were higher in the non-abstainers (Table 6). The BMI and the prevalence of pregnancy hypertension were lower in the non-abstainers. German women were overrepresented in the group of non-abstainers whereas women with a non-German background were mostly abstainers (p < 0.001). Prevalence rates were 9.5% for SGA and 6.2% for PTB (Table 6).

**Table 6:** KiGGS study: sample characteristics (N = 16301) according to the level of alcohol intake during pregnancy

	**Total N = 16301**	**Abstainer N = 14089 (86.4%)**	**Non-abstainer N = 2212 (13.6%)**	**P for trend**
Maternal education (%)				
Low	23.5	24.7	15.8	<0.001
Middle	47.4	48.0	43.9	
High	29.1	27.3	40.0	
Smoking during pregnancy (% yes)	17.0	16.6	19.5	0.001
Maternal height (cm)	166.5 (6.3)	166.4 (6.3)	167.4 (6.2)	<0.001
Maternal BMI	24.5 (4.7)	24.6 (4.7)	24.2 (4.6)	<0.001
Maternal age (years)	28.2 (5.1)	28.0 (5.1)	29.4 (5.0)	<0.001
Parity (% >0)	67.9	67.6	69.9	0.044
Ethnicity (%)				
German	84.6	83.5	91.5	<0.001
Non-German	15.4	16.5	8.5	

Data were missing for maternal education (N = 448), smoking during pregnancy (N = 89), maternal height (N = 188), maternal BMI (N = 299), maternal age (N = 154), parity (N = 3457), ethnicity (N = 113).

Values are percentages for categorical factors, or means (with standard deviations) for continuous factors.

In both, the univariate and multivariate regression, maternal alcohol consumption was not related to SGA: adjusted OR for alcohol exposure was 0.98 (95% CI 0.81, 1.19) (Table 2). On the other hand, an inverse relationship was observed between alcohol consumption and the risk of PTB. After adjustment for confounders, the association between prenatal alcohol exposure and PTB was significant with an OR of 0.75 (95% CI 0.57, 0.99) (Table 2).

The interaction term between alcohol intake during pregnancy and maternal education was not significant (p-values for interaction ranged from 0.399 to 0.625). The same applied to smoking during pregnancy (p-values for interaction ranged from 0.283 to 0.424). Stratified analyses by educational level or by smoking did not provide evidence to suggest that the effects of alcohol would be greater in the offspring of low educated mothers or mothers who smoked during pregnancy (Tables 3 and 4).

## Discussion

### Key findings

Contrary to our expectations, we found that the risk of SGA and PTB was not increased in the offspring of mothers who consumed alcohol at any frequency (daily, non-daily or at some point) during pregnancy. SGA was not associated with alcohol intake during pregnancy, while PTB was found to be inversely (instead of positively) related. The associations mentioned above did not differ according to levels of maternal education, high levels of distress, or tobacco intake during pregnancy. Stratified analyses showed no adverse effects of low-moderate alcohol intake on SGA and PTB, not even in the offspring of women who were disadvantaged in terms of low education, high levels of distress, or tobacco intake during pregnancy.

### Evaluation of potential limitations

First, selective participation may have occurred with an inclusion of mainly healthy females and a higher non-response in women with a heavy alcohol intake. Therefore, our results only apply to light-moderate alcohol intake and cannot be generalised to women with heavy alcohol intake during pregnancy.

Second, alcohol intake during pregnancy was measured by self-reports, which are known to underestimate the frequency and amount of alcohol intake of pregnant women [47-50]. Furthermore, we did not assess whether non-daily drinkers were binge drinking during pregnancy at a certain point. Underestimation and misclassification of alcohol consumption may have contributed to the lack of evidence concerning an adverse effect of alcohol intake on SGA or PTB.

Third, neither of the studies measured in detail the amount of alcohol intake during different phases of pregnancy. The first trimester is considered to be a highly ‘alcohol vulnerable’ period in fetal development [51]. Due to lack of data on the timing of alcohol intake, we might have missed the adverse effects of alcohol intake in this specific period.

Fourth, in the KiGGS study, information on SGA and PTB was derived from the parents’ questionnaire up to 17 years retrospectively. Although the prevalence rates of SGA and PTB are similar to those in the ABCD study, recall error and self-reporting bias is likely to have occurred, which could affect the associations between alcohol intake during pregnancy and the pregnancy outcomes in unknown ways.

### Discussion of the key findings

In both studies we found the risk of PTB to be significantly decreased among children of mothers who drank during pregnancy. Our findings are compble with others that report an inverse association between alcohol intake and the risk of PTB [14,17,22], and to studies which report a J-shaped association between alcohol consumption and the risk of PTB [3,10]. A decreased risk of non-daily drinking was also observed in a Danish study that suggests a decreased risk in the offspring of women who consume 2–4 drinks per week [3], and an Australian study that reports no adverse effect of up to six glasses per week [16]. While our findings contrast to the findings of one Dutch study [8], another study from the Netherlands also found that PTB was lower in those who consumed alcohol with up to 120 grams per week and more [22]. Another study from Denmark suggested a decreased risk of PTB with up to 9 glasses per week [10]. Both studies from Denmark report a threshold of an increased risk at ≥ 10 and more glasses per week and ≥ 4 glasses per week, respectively [3,10]. Binge drinking was also reported to result in adverse effects for PTB [16]. Unfortunately, we cannot report on the association between PTB and binge drinking or heavy daily alcohol intake as we have only 29 cases of women who reported more than one drink per day and we did not collect information on binge drinking.

An explanation for our finding of the reduced risk of PTB might be the contradiction-inhibiting effect of alcohol, which reduces the release of the birth hormones vasopressin and oxytocin [52-54]. Until the 1970s, alcohol was even used in tocolysis because of its positive effect on reducing the risk of PTB [55-57]. However, the findings on the usage of alcohol to treat threatening PTB are controversial [57-61]. In addition, the evidence for a positive effect is weak, as randomized control trials showed no effect of alcohol to prevent PTB [59-61] and the side-effects of alcohol were considered to be unacceptable [21,60]. Thus, this treatment method was abandoned.

Another explanation for the observed inverse association with PTB might be residual confounding. Pregnant women who continue to consume alcohol might be healthier in many unmeasured terms, such as general state of health, healthier nutrition, and living conditions etc. (the ‘healthy drinker effect’) [62].

In both studies we found no association between any level of alcohol intake (daily, non-daily, non-abstaining) during pregnancy and SGA. These findings are compble to another study from the Netherlands, suggesting no effect of alcohol ≥ 120 grams per week on birth weight [22]. Similar results were reported in an Australian study indicating that < 7 drinks per week and not more than 2 standard drinks per occasion are not associated with SGA [16]. Our findings are also similar to a study from Italy showing no increased risk of SGA with up to 2 drinks per day [6]. However, a study from Australia [16] and one from Italy [6], as well as two studies from the USA [19,23] report increased risks of SGA in women with heavy drinking (> 2 drinks per day) and bingeing patterns. Though our results do not suggest a negative association with alcohol intake in general, we cannot exclude the possibility of an association with high levels of daily alcohol intake or binge-drinking.

Contrary to our initial hypothesis, there was no interaction between alcohol intake during pregnancy and maternal education. Alcohol intake during pregnancy was not associated with adverse effects in the offspring of lower educated women. This finding is in contrast to studies on FAS, where especially the offspring of lower educated women were found to be vulnerable to the adverse effects of alcohol [25-29]. In contrast to patient studies on FAS, our sample does not include severely alcohol-addicted women, since only 0.5% of the women reported a consumption of more than one standard drink per day.

In contrast to what we expected, and in contrast to others [5,22,31,32], the results from both these studies show no increased risks of PTB and SGA in women who both smoked and drank, compared to drinkers alone. However, our finding of no interactive effect between alcohol and smoking on birth weight concurs with results reported from Finland [9] and France [12]. However, more research is needed in other study populations to elucidate the interactive mechanism and to detect a possible threshold of alcohol and smoking on pregnancy outcomes.

In women with high levels of mental distress, the positive effect of alcohol on PTB disappeared. Animal studies have shown that exposure to prenatal alcohol and distress together has a more adverse effect on birth weight than the single exposures [63]. However, although the mediating effects remain unclear [63], animal studies showed that stress causes increased release of hormones that interact with alcohol that affect birth weight and brain development [36,37]. As this is the first study in humans to investigate a possible interaction between prenatal alcohol and distress on PTB, our findings need to be reproduced in other human studies.

## Conclusions

Our findings are in agreement with many others in suggesting that there is no increased risk for SGA and PTB in the offspring of mothers who consume low to moderate quantities of alcohol during pregnancy. This applies to both the studies, although it should be emphasised that the results of the KiGGS study might be affected by recall bias. We observed no adverse effects of low-moderate alcohol intake in the offspring of women who were disadvantaged in terms of low education, high mental distress or tobacco intake during pregnancy. Further studies in disadvantaged populations are needed to replicate our findings.

## Abbreviations

ABCD: Amsterdam born children and their development; ANOVA: One-way-analysis of variance; BMI: Body mass index; CAPI: Computer assisted personal interview; CES-D: Center for epidemiological studies depression scale; CI: Confidence interval; FAS: Fetal alcohol syndrome; KiGGS: German health interview and examination survey for children and adolescents; OR: odds ratio; PTB: Preterm birth; SES: Socioeconomic status; SGA: Small for gestational age; SPSS: Statistical package of social sciences; STAI: State-trait anxiety inventory.

## Competing interests

The authors declare that they have no competing interest.

## Authors’ contributions

AK, MP and TV designed the study. MP analysed the data and wrote the manuscript. AK, RF and TV contributed to the interpretation of the data and revised drafts of the manuscript. ME contributed to the ABCD study design and the data collection. All authors commented on interim results and drafts. All have read and approved the submission of the manuscript.

## Authors’ information

MP is a PhD student at Bielefeld University, Germany. AK is an associate professor at the Department of Public Health at the Academic Medical Center, part of the University of Amsterdam, the Netherlands. RF is a physician at the University Hospital Munster, Germany. ME is a senior epidemiologic researcher and project leader of the ABCD-study at the Public Health Service (GGD) Amsterdam, the Netherlands. TV is project leader of the ABCD study and assistant professor at the Department of Public Health at the Academic Medical Center, part of the University of Amsterdam, the Netherlands.

## Pre-publication history

The pre-publication history for this paper can be accessed here:

http://www.biomedcentral.com/1471-2393/13/49/prepub
